# A Defence of Cocaine in Cataract Extractions in Answer to Dr. Calhoun’s Article

**Published:** 1886-10

**Authors:** J. M. Hull

**Affiliations:** Augusta, Ga.; Special Professor of Eye, Ear and Throat Diseases in the Medical Department of University of Georgia, Etc.


### A DEFENCE OF COCAINE IN CATARACT EXTRACTIONS IN ANSWER TO DR. CALHOUN'S ARTICLE.

BY J. M. HULL, M. D., SPECIAL PROFESSOR OF EYE, EAR AND THROAT DISEASES IN THE MEDICAL DEPARTMENT OF UNIVERSITY OF GEORGIA, ETC.

There appeared in the March (1886) number of THE ATLANTA MEDICAL AND SURGICAL JOURNAL a clinical lecture by Dr. A. W. Calhoun, on cataract extractions, in which, with reference to the use of anæsthetics, he says:

"My opinion as to the use of cocaine in cataract extractions has undergone recently a complete change.

"It relieves the pain of the operation absolutely, and quiets the fears of the most timid patients, but the results in many instances are anything but satisfactory.

"The use of even a weak solution of cocaine causes decided contraction of the conjunctival and sub-conjunctival blood-vessels, and this diminishes the amount of nutrition to the cornea.

"When the wound is made for the removal of the lens the cocaine comes in contact with the cut surface of the cornea and also passes into the anterior chamber, and in the course of twelve to twenty-four hours violent inflammation, with more or less sloughing of the cornea, ensues. Even where this does not occur, iritis is very apt to follow with very profuse effusion of lymph in the pupillary space and posterior chamber. For these reasons I advise you against the use of cocaine in cataract extractions. The operation is not a very painful one, and there are very few patients who will not gladly undergo the operation with the expectation of having their sight restored."

In a paper, entitled "The Other Side of Cocaine—The Bad Side," read before the State Medical Association in April, the doctor further stated, "That before he began the use of cocaine from 95 to 97 per cent. of all cataract extractions proved successful, and that in a large number, where cocaine had been used, he had bad results. He has abandoned its use altogether in operations by extractions."—ATLANTA MED. AND SUR. JOURNAL, p. 195.

My own experience with cocaine in the extractions of cataract had been so totally different from Dr. Calhoun's that I read with amazement the statement of his results.

Decided contraction of the conjunctival and sub-conjunctival blood-vessels is certainly produced by the use of cocaine, but the cornea is not so dependent upon these for its nutrition as to allow sloughing to occur in consequence of this contraction, which lasts at the longest but a few minutes.

The effect upon the iris is to diminish its blood supply and also to dilate its pupil, so I can't believe that inflammations of that membrane can justly be ascribed to cocaine.

Up to the time of reading the doctor's clinical lecture, I had used cocaine in 23 extractions and over 60 iridectomies, and without a single bad result, and since then have used it in nine cases, in one of which both eyes were operated upon at one sitting (that being the fourth time I have removed both cataracts at once successfully), and in only one case have I had any inflammatory trouble.

In this case it was an iritis, and caused undoubtedly by the great difficulty experienced in removing the lens, it being partially dislocated, and the consequent injury done that sensitive structure.

I have not found more than four drops of a 4 per cent. solution of cocaine necessary to produce the desired anæsthesia, (though in the beginning I have often used more), and the lids should be kept closed over the cornea until the eye is sufficiently insensible to operate upon, when the speculum should be introduced and the operation completed as rapidly as possible. The cleansing of the eye can be done after the speculum has been removed and the anæsthetized cornea again afforded the proper protection.

Delicate handling during an operation and the utmost care afterwards have always been recognized as absolutely necessary to a normal recovery after cataract extraction, and I should look to the absence of these should my operation be attended with inflammatory reactions.

Thorough care should be taken of instruments, and none save the surgeon be allowed to handle them, and he only when his hands and clothes are antiseptically clean.

From the careful teachings of my step-father, Dr. DeSaussure Ford, I early learned that the surest way to avoid suppurative troubles with wounds was to put your patients under the influence of quinine and then preserve absolute cleanliness with your dressings.

Five years' experience in extensive eye surgery has taught me that the principles taught and practiced by him in general surgery are equally applicable in operations and wounds of the eye.

Therefore, in preparing for the operation, in addition to giving a mild cathartic the night before, 5 grains of quinine are also given, which should be repeated at 8, 2 and 8 on the day of operation and on the day following.

In 1876, in a paper read before the Georgia Medical Association, Dr. Ford, in a "Report of surgical cases occurring in his practice," first called attention to the value of administering quinine in cataract extractions. Dr. A. S. Campbell, in 1880, also reported on the same subject.

In 1881 Dr. Ford, again writing on "Quinine and Tar Water as

Antiseptics," gives the following quotation from Dr. Robert Campbell, to whom the honor is due for his study of quinine and recommendations for its intelligent use in recent times.

He says: "Quinine acts upon the fibrous coat of the vessels, constringing them, and thus preventing congestions." He remarks further, "that it is a disseminator or equalizer of the circulation, and acts by dispersing, wherever found, all vascular accumulations, possibly by giving tone to the vascular tissue, and that it has control over the nervous system (vaso-motor) under such circumstances by dispersing such engorgements in its centres."

His son subsequently writes: "Quinine restores the proper tone of the vessels, enabling them to contract and regain their normal calibre, when stony and consequently blood stases exists, relieving hyperæmia and engorgements, and thus preventing, limiting or relieving inflammation."

Accepting these views, it naturally follows that when quinine is administered freely, and the system under its influence at time of operation, inflammatory troubles most rarely result, and by carrying out these precautions as prophylaxis, I have been able to give my patients the great benefits of cocaine anæsthesia, and yet been entirely free from inflammatory reactions.

The other details, as to the use of atropia, manner of dressings, etc., are those practiced by all careful oculists and require no mention here.

I am not, however, in sympathy with the practice that requires patients to be confined to their beds in a very dark room for twelve or fourteen days, as thereby they become despondent, restless, and in many instances debilitated. Therefore, when everything is progressing nicely, I allow them to sit up on the second or third day, if they desire it, and do not find recovery retarded thereby.

In elderly persons, and in those not in robust health, whisky should be given two or three times a day during the first few days after the operation.

In conclusion I will say that cocaine is the most valuable anæsthetic yet known, and can be used in cataract extractions, as wel as all other operations on the eye, with absolute relief from pain, and in combination with quinine with perfect freedom from inflammatory reactions.

